# SMDFbs: Specification-Based Misbehavior Detection for False Base Stations

**DOI:** 10.3390/s23239504

**Published:** 2023-11-29

**Authors:** Hoonyong Park, Philip Virgil Berrer Astillo, Yongho Ko, Yeongshin Park, Taeguen Kim, Ilsun You

**Affiliations:** 1 AUTOCRYPT Co., Ltd., Seoul 07241, Republic of Korea; hypark@autocrypt.io; 2 Department of Computer Engineering, University of San Carlos, Cebu 6000, Philippines; pvbastillo@usc.edu.ph; 3 Department of Financial Information Security, Kookmin University, Seoul 02707, Republic of Korea; koyh0911@kookmin.ac.kr (Y.K.); p17030508@kookmin.ac.kr (Y.P.); 4 Department of Information Security Engineering, Soonchunhyang University, Asan 31538, Republic of Korea; tg.kim@sch.ac.kr

**Keywords:** false base station, 5G, intrusion detection system, specification-based detection, machine learning

## Abstract

The advancement of cellular communication technology has profoundly transformed human life. People can now watch high-definition videos anytime, anywhere, and aim for the implementation of advanced autonomous driving capabilities. However, the sustainability of such an environment is threatened by false base stations. False base stations execute attacks in the Radio Access Network (RAN) of cellular systems, adversely affecting the network or its users. To address this challenge, we propose a behavior rule specification-based false base station detection system, SMDFbs. We derive behavior rules from the normal operations of base stations and convert these rules into a state machine. Based on this state machine, we detect network anomalies and mitigate threats. We conducted experiments detecting false base stations in a 5G RAN simulator, comparing our system with seven machine learning-based detection techniques. The experimental results showed that our proposed system achieved a detection accuracy of 98% and demonstrated lower overhead compared to other algorithms.

## 1. Introduction

5G technology has been extensively researched with the primary objective of achieving faster speeds, lower latency, and the ability to support a higher number of concurrent connections when compared to LTE [1]. In order to enable these improvements, 5G has introduced a fundamental architectural shift by separating the Distributed Unit (DU) from the Centralized Unit (CU). This separation lays the foundation for delivering services with ultra-low latency communication, which is crucial for applications like smart buildings, vehicle-to-everything (V2X) communication, and autonomous driving [2]. However, despite these remarkable advancements in mobile communication technology, the threats landscape remains dynamic and challenging. One persistent concern is the risk of false base station attacks, where malicious actors deploy deceptive base stations that mimic legitimate ones, often with the intention of stealing user information or launching resource depletion attacks such as Denial of Service (DoS). Consequently, ongoing research efforts are dedicated to devising effective strategies and countermeasures to mitigate these threats. Effective security measures ensure reliability, and sustainability of 5G environment and the services it offers [2].

A false base station attack takes advantage on the common behavior of User Equipment (UE), which tends to connect to the strongest signal available. The exploit involves broadcasting a stronger signal to lure the UE into connecting, after which various active and passive attacks can be initiated [3]. These attacks include, but are not limited to, Authentication Relay attack, International Mobile Subscriber Identifier (IMSI) Catching, and Denial of Service (DoS) attacks. As these tactics pose ongoing security challenges, sustained research efforts are imperative to develop effective countermeasures and safeguard against these threats [3].

In response to these persistent threats, countermeasures against false base stations have improved alongside the advancement of mobile communication technologies. While LTE transmitted in the plaintext the user’s unique identifier, the IMSI, 5G has significantly enforce security by encrypting the user’s unique identifier, now referred to as Subscriber Permanent Identifier (SUPI), transforming it into the Subscriber Concealed Identifier (SUCI) for secure transmission [4]. While the encryption of user identifiers in 5G encompasses a significant increase in cost for potential attackers to compromise user privacy in contrast to LTE [5], the persistent concern of security threats stemming from false base stations remains a challenge within the 5G landscape. Numerous detection techniques have been proposed to mitigate these threats, spanning a range of methodologies such as cryptography-based [6], rule-based [7], machine learning-based [8,9,10], and Radio Frequency (RF)-based methodologies [11]. However, it is worth noting that cryptography methods may introduce additional expenses related to group keys and certificate management. Moreover, though a Message Authentication Code (MAC) is employed, there remains the risk of replay attacks by malicious actors, creating challenges for UEs in accurately discerning between a legitimate base station and fraudulent one. Furthermore, some research endeavors have faced challenges in tailoring their solutions to the distinctive feature of the 5G system or in formulating practical deployment strategies. This highlights the imperative for innovative and adaptable approaches to ensure security and sustainability of the 5G network ecosystem [11].

In this paper, we present a novel false base station attack detection system, leveraging behavior rule specification-based method tailored to the 5G ecosystem. Our proposed innovative approach involves the derivation behavior rules that govern the behavior of UEs under normal conditions. This process as base stations handle the traffic generated by UE actions in the 5G mobile communication environment. The proposed method culminates in the formulation of a finished state machine, which defines whether the UE is misbehaving or not. This method is notably lightweight when compared to resource intensive contemporary methods such machine learning-based detection techniques. Furthermore, the specified behavior rule-based false base station attack detection system has the capability to detect ongoing attacks in real-time by executing the finite state machine. This presents a distinct advantage, as it enables both users and mobile operator respond promptly and effectively previously unknown attacks [3].

Furthermore, to assess the effectiveness we have expanded upon the capabilities of the 5G Radio Access Network (RAN) simulator, specifically utilizing ALİ GÜNGÖR’s UERANSIM version 3.2.6. Accordingly, the mobility features for UE has been integrated and implemented functional segmentation of the gNodeB (gNB). This functional segmentation splits the network into CU and DU, a strategic approach designed to optimize latency within the 5 GB K. Furthermore, we have incorporated the 3GPP Propagation model to enable seamless wireless signal strength measurements. Within the simulation, UEs dynamically move around a predefined areas and execute Inter gNB-DU Handover [12,13]. The key contributions of this research are as follows:

- We introduced an innovative false base station attack detection technique founded on behavior-rule specification-based approach. In contrast to detection methods, such as machine learning-based techniques require prolonged learning period and impose heavy computational burden, our proposed approach relies on accurate system specifications as basis in systematically defining the policies or rules. This method significantly eliminates machine learning time and provides an efficient and robust security solution.
- We enhanced the 5G RAN simulator, UERANSIM, within the 5G ecosystem by implementing a structural approach known as Functional split. Additionally, we incorporated the 3GPP Propagation model to construct a simulation environment that facilitates seamless Inter gNB-DU Handover.
- We executed a state machine using experimentally generated data to evaluate the performance and overhead. We demonstrated the effectiveness of the proposed method through comparative analysis with seven (7) contemporary machine learning algorithms.

The remainder of this paper is structured as follows: Section 2 offers insights into the structure of 5G RAN, elucidates security threats emanating from base stations, and elaborates on the behavior-rule specification-based technique for detection abnormal behaviors. Subsequently, Section 3 provides a in-depth review of existing research endeavors centered around the false base station detection, and Section 4 presents the proposed technique. In line with this, Section 5 describes the experimental environment employed to validate the proposed technique, along with comprehensive analysis of the obtained results. Finally, Section 6 concludes this paper and outlines future research directions.

## 2. Background and Security Threats

### 2.1. 5G Architecture

**Figure 1:**
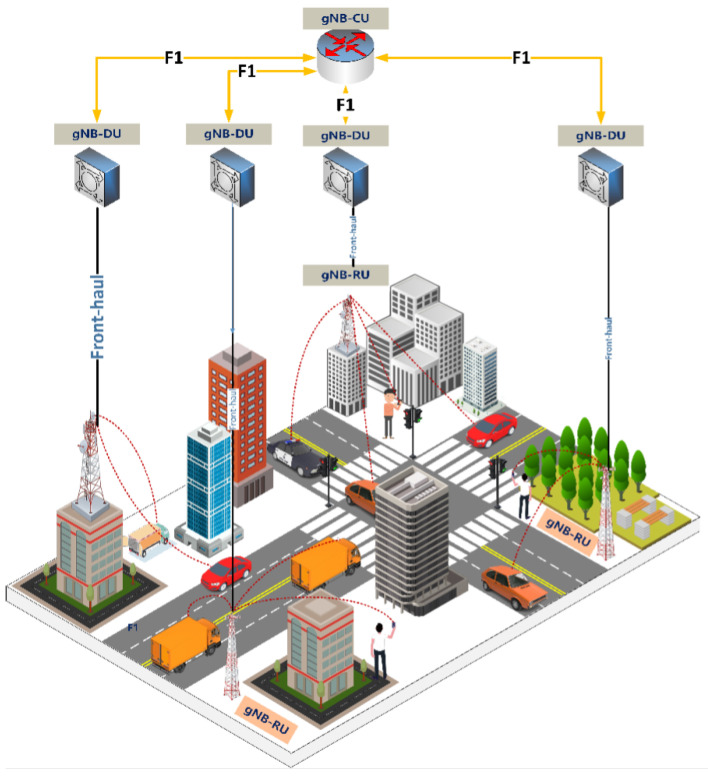
NG-RAN architecture.

Figure 1 illustrates the general architecture of 5G, with a particular focus on the Next-Generation Radio Access Network (NG-RAN), serving as the point of connection for UEs to the 5G Core Network. Meanwhile, the unique characteristics of 5G wireless frequencies within the millimeter wave spectrum, characterized by high directivity with shorter signal coverage, necessitates the deployment of base stations in a more densely distributed manner compared to its wireless network predecessors. In addition, this challenge is mitigated through function split in NG-RAN, such that the gNB functions were separated into three distinct entities: the Central Unit (CU), Distributed Unit (DU), and Radio Unit (RU). This architectural innovation plays a pivotal role in enhancing the efficiency and scalability of 5G networks, enabling them to effectively address the coverage limitations posed by the millimeter wave spectrum.

CU establishes connections with the Access and Mobility Management Function (AMF) and User Plane Function (UPF) of the 5G Core network. It is build upon the foundation of LTE’s eNodeB (eNB). Within its domain, CU manages critical functions, including Packet Data Convergence Protocol (PDCP), Radio Resource Control (RRC) layers, and their upper layers. DU, on the other hand, take on the responsibility of facilitating data communication tunneling between User Equipment (UE) and CU, ensuring seamless data flow within the network. The RU stands as the physical component, performing a crucial role in the conversion of digital signals received from DU into RF signals. It processes wireless signals for transmission and reception through antennas. Notably, this architecture allows for the connection of multiple DUs to a single CU, with each DU linked to a corresponding RU. This strategic design enables cost-efficiency, scalability, and virtualization advantages [14].

### 2.2. Security Threat from a False Base Station

A false base station refers to an unauthorized base station capable of launching attacks on User Equipment (UE) or the mobile communication network. These deceptive entities exploit the the common behavior observed in UEs, which is the tendency to connect to stronger wireless signals. False base stations manipulate this inclination by emitting amplified signals, overpowering the legitimate base stations in their vicinity. In consequence, UEs are coerced into connecting to these rogue stations. Additionally, to increase the success rate of their attacks, false base stations mimic the appearance or characteristics of legitimate ones. Attackers employ false base stations to launch various types of attacks, with one of the most notorious among them being the IMSI-Catcher [15] This attack represents a significant security threats, and it is widely recognized as a formidable risk to users and the network alike.

One of the most well-known attacks, namely IMSI-Catcher, involves enticing nearby UEs into establishing a connection. This attack enables the interception of IMSIs transmitted in plaintext, thereby posing a substantial risk to the privacy of subscribers [16]. Once in possession of these collected IMSIs, attackers can exploit them to track the location of subscribers, further compounding the threat to user’s privacy [17].

Moreover, malicious actors can launch attacks that target system availability. The second attack worth noting is the Denial of Service (DoS) attack, where false base stations obstruct communications between UEs and legitimate base stations by acting as intermediaries in the communication flow. This obstruction leads to the occurrence of complete or partial DoS attacks, severely disrupting the normal operation of the network.

Lastly, threat actors can resort Radio Access Technology (RAT) downgrade attacks. These attacks are designed to prevent Tracking Area Update procedures, effectively forcing the network to downgrade to a lower generation than the one it currently supports and to which the UE is connected.

In addition to aforementioned attacks, it is also worth noting that false base stations can also have a detrimental impact on mobile communication networks. These environments are often dependent on Self-Organizing Network (SON) functions, which play a significant role in network self-configuration, optimization, and recovery. At the highest network level, SON functions capitalize Measurement Report (MR) messages sent by UEs. Unfortunately, within this context, UEs lack the capability to distinguish between wireless signals transmitted by false base stations and those emanating from legitimate ones. This vulnerability can lead to inclusion of signals originating from false base stations in the MR messages. Such an occurrence has the potential to disrupt SON functions and opens opportunities for malicious actors to manipulate UE location information.

While collecting IMSIs is much more challenging in 5G context, attackers still have avenues for exploitation. They can target unencrypted unicast messages or intercept and retransmit broadcast messages from legitimate base stations. Furthermore, attackers can execute a variety attacks, including Denial of Service through man-in-the-middle attacks, SON manipulation, and tampering with location information. These threats can carry substantial implications for network availability and may present heightened security risks in the context of ultra-low-latency services. Therefore, safeguarding against these vulnerabilities remains a paramount concern in the evolving landscape of 5G network security.

## 3. Related Works

### 3.1. Detecting False Base Stations

The emergence of new false base station detection techniques has enhanced the security of the existing mobile communication environment. However, security threats have continued to persist. Consequently, the need for enhancing security has been an ongoing issue, particularly in the context of detecting false base stations and mitigating threats.

As part of these primary efforts, various approaches have been proposed to identify false base stations through physical information. Ali et al. proposed a scheme where UE can detect false base stations through Radio Frequency (RF) Fingerprinting of legitimate base stations [11]. Li et al. introduced the FBS-Radar system, which collects and analyzes received spam messages and metadata from UEs to detect the location of false base stations [18]. Bin et al. proposed a method for identifying false base stations through signal strength analysis using the well-known clustering algorithm Density-Based Spatial Clustering of Applications with Noise (DBSCAN) [19]. Shin et al. presented a whitelist detection approach using the Automatic Neighbor Relation (ANR) procedure to confirm the Physical Cell Identifier (PCI) values of legitimate base stations and detect PCI duplication, thereby preventing forgery of legitimate base station information [20]. However, research on identifying false base stations through physical information lacked experimental results for various scenarios, such as noise interference, and was simulated in the previous generation of mobile communication environments, which posed challenges for applying these techniques in the 5G system.

Additionally, Park et al. proposed the Broadcast Message Authentication Network Function (BMANF) protocol, which enables UEs to ignore messages received from false base stations or avoid establishing connections with them by generating MACs based on group keys or public keys in the core network, base stations, and UEs [6]. However, cryptographic techniques required additional costs related to group key and certificate management, and attackers can obtain the generated MAC and retransmitted it. In this point, the identification of false base stations had obvious limitations.

Furthermore, Nakarmi et al. proposed a network rule-based false base station detection technique to identify cell identifiers that are compatible with 3GPP Radio Access Technologies (RATs) but unauthorized [7]. However, there was a lack of specific explanations for identifying false base stations that masqueraded as legitimate base stations in the same location, and the experiments were conducted in an LTE environment, limiting their applicability to the 5G system.

Lastly, machine learning-based false base station detection techniques have been proposed. Nakarmi et al. proposed a machine learning-based detection method based on Reference Signal Received Power (RSRP) [8], and Jin et al. proposed an approach using the well-known machine learning algorithm, lightGBM, to identify false base stations based on base station parameters [9]. However, both approaches were tested in previous generation mobile communication environments, and have limited application to the 5G system. Park et al. created false base station classification models using four machine learning algorithms (SVM, KNN, Decision Tree, Naive Bayes) in the 5G system. They used these models for detection and conducted a performance evaluation by comparing the performance of each algorithm [10]. The machine learning-based false base station detection technique proposed in their paper was tested in the 5G system. However, it showed limitations in detection when attackers broadcast signals with similar signal strength as legitimate base stations from the same location. While machine learning-based detection techniques demonstrated the most robust detection performance among the proposed methods, but they required high implementation costs, required a long training period for accurate machine learning-based detection, had to rely on MR, and imposed significant overhead. Therefore, it was evident that real-time detection posed a burden.

### 3.2. Anomaly Detection Based on Behavior Rule Specifications

The Anomaly Detection algorithm is effective in countering unknown attacks, including zero-day attacks. However, it often imposes a significant computational overhead and requires substantial costs related to profiling and learning abnormal patterns. In contrast, the approach proposed in this paper, which is based on behavior rule specification, demands expertise in defining specifications but offers a lower cost while achieving high detection accuracy and low false positives and false negatives. By using behavior rules, this approach effectively detects zero-day attacks. Additionally, since it leverages a specified finite state machine, it is well-suited for low-cost implementation in resource constrained IoT embedded environments, where computing power is limited.

This behavior rule specification-based detection technique has been researched in various contexts. Sharma et al. developed the BRIoT tool and applied it in Unmanned Aerial Vehicle (UAV) environments, using a state machine validated through the application of a two-layer Fuzzy-based Hierarchical Context-aware Aspect-oriented Petrinet (HCAPN) [21]. Astillo et al. proposed the TrMAps scheme and applied it to the Artificial Pancreas System (APS) to detect specification-based abnormal behaviors in the controller, Continuous Glucose Meter (CGM), and insulin pump [22]. Unlike Sharma et al., they used the UPPAAL tool for formal verification of the state machine [23].

In this paper, behavior rules were derived based on system requirements and security threats in a given environment to create behavior rules for precise specification. These generated behavior rules were then transformed into state transition conditions for a state machine with two states: normal and abnormal, within the anomaly detection system. Subsequently, formal verification was performed using the UPPAAL tool to ensure the proper creation of the state machine. If the verification succeeds, the malfunction detector takes input data from one or more components, runs the state machine, and determines whether the current system state is normal or abnormal. To reduce false positives and false negatives in the decision-making process, compliance is applied to assess whether the system is truly normal or abnormal based on the decision outcome.

## 4. Proposed System

**Figure 2:**
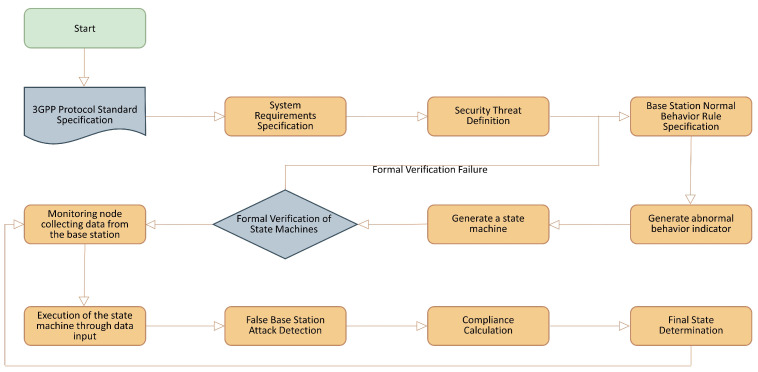
Sequence diagram for the proposed specification-based false base station attack detection technique.

The proposed system extracts system requirements and security threats from the 3GPP standard specifications and defines normal behavior rules based on security aspects. Using these normal behavior rules, a state machine is generated and formally verified. After verification, false base station attacks are detected through monitoring. This process is illustrated in Figure 2.

### 4.1. Adversarial Model

The attacker uses a false base station to send signals that are equal to or stronger than those transmitted by adjacent base stations, enticing the User Equipment (UE) to connect. When the UE attempts to connect, the attacker either carries out a passive attack or an active attack. They modify the unencrypted UE_Capability_Information message exchanged between the UE and next-generation NodeB (gNB) to prevent the UE from receiving appropriate radio services or they retransmit the RRC_Setup_Request message to break the Radio Resource Control (RRC) connection of the UE. Additionally, by blocking message delivery, they induce a denial-of-service attack. While the attacker possesses in-depth knowledge about the 3GPP standard protocol, without the keys used for encryption, they cannot decrypt encrypted content. Similarly, they cannot reverse a one-way hash function or guess random numbers.

### 4.2. Derivation of Normal Behavior Rules

**Figure 3:**
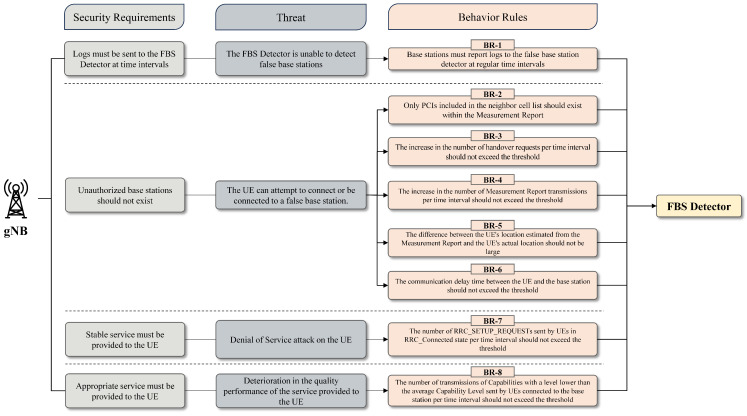
Process of deriving behavior rules for the base station.

Behavior rules form the foundation of software agents that determine the state of trustworthy components in a system. In this paper, the derivation of behavior rules is based on the 3GPP standard specifications. Before deriving behavior rules, a security context pertaining to system requirements is applied to establish a strict sense of how base station components should operate from the perspective of a false base station detection agent. As a result, the implementation of such a context leads to the establishment of security requirements for the base station. During the composition of each security requirement, all potential threats that could hinder the system from meeting the said requirements are identified, regardless of the severity or exploitability of such threats. Once threats are identified, they aid in deriving more realistic and meaningful behavior rules, in conjunction with confidentiality, integrity, and availability. Figure 3 illustrates the sequential steps in deriving behavior rules for the base station.

### 4.3. Design of SMDFbs Software

**Figure 4:**
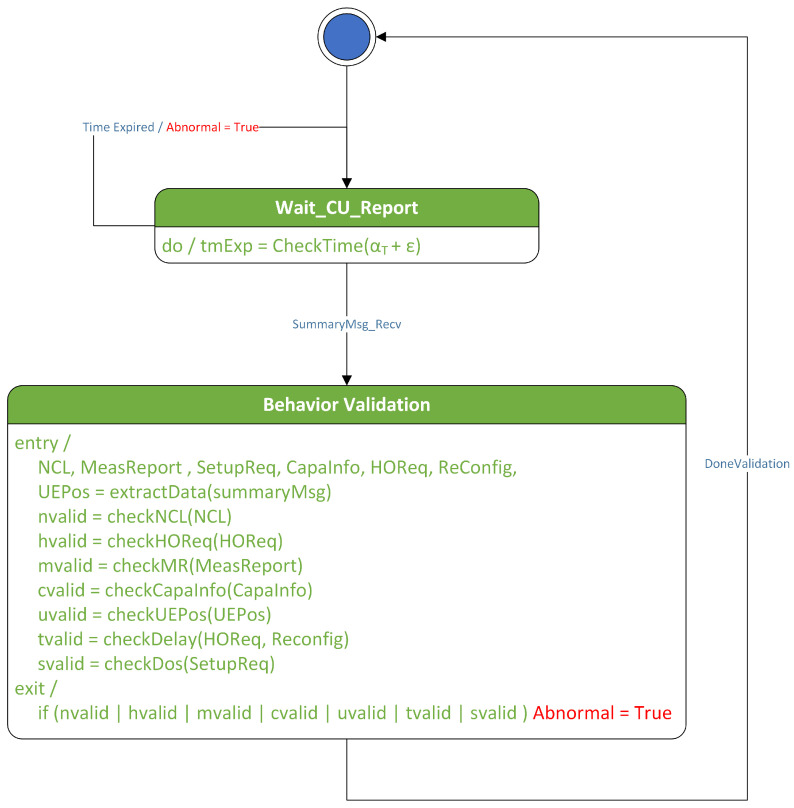
UML state diagram of SMDFbs.

In the proposed technique, behavior rules are implemented as a state machine to be executed in the Specification-based False Base Station Attack Detection System (SMDFbs). This state machine collects portions of the RRC message logs exchanged between the base station and the UE, as well as the handover request history. The messages collected include the RRC_Setup_Request message, UE_Capability_Information, and the Measurement Report. A software model is a way to represent software design. The commonly used method is Unified Modeling Language (UML). In this paper, the UML state diagram is used to represent the software design of SMDFbs. Figure 4 illustrates the SMDFbs represented as a UML state diagram.

### 4.4. Verification of SMDFbs Software Design

A state diagram represents the sequence of events in the base station and the derived behavior rules, making it essential to formally verify the functional accuracy of the model before software development. Such verification ensures that the derived behavior rules are fully covered; otherwise, a high rate of undetection may occur. To verify the state diagram of SMDFbs, we use UPPAAL, an integrated environment tool for system modeling, simulation, and verification, for formal verification [23]. UPPAAL model checking performs state space exploration, allowing users to evaluate the required specifications by defining computational tree logic (CTL).

First, to validate the state machine tailored to the given environment, we represented the state changes in UE, DU, and CU in the Inter-gNB-DU handover scenario through a state diagram. As previously described, we abstract and model each entity’s state diagram using the UPPAAL model checking tool.

**Figure 5:**
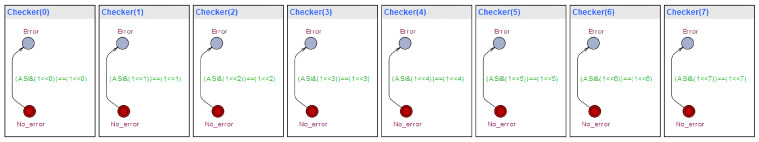
ASI reachability verification.

The properties verified through UPPAAL include reachability and safety for all behavior rules. Figure 5 shows the UPPAAL template that checks whether an abnormal state has been reached, deviating from the defined behavior rules. Here, the Attack State Indicator (ASI) is an indicator used to check whether each behavior rule has been violated.

**Figure 6:**
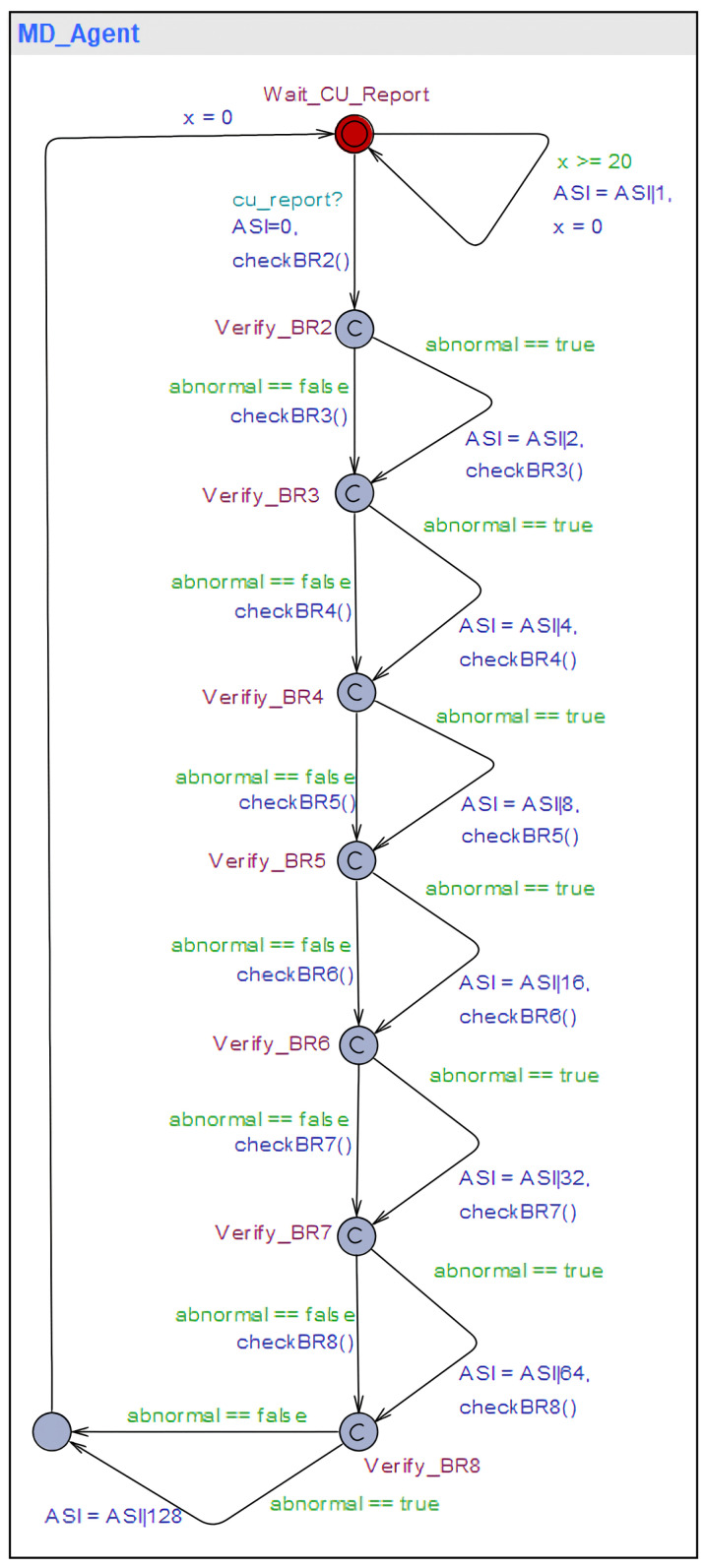
UPPAAL model of SMDFbs.

Figure 6 represents the abstraction of SMDFbs in the UPPAAL model verification tool. Since it abstracts the code that was executed in the experiment for UPPAAL model verification, there are differences from the actual implementation code.

Table 1 verifies the properties of the model based on reachability analysis. Queries about the properties are expressed in Computation Tree Logic (CTL). The model’s safety was verified by querying whether the system falls into a deadlock. The reachability of each behavior rule was verified to understand that it does not reach when normal but reaches when abnormal.

### 4.5. Compliance Statistical Analysis

The defined compliance is one of the main metrics for measuring the safety and reliability of a system and is a key metric for detecting abnormal behavior of the proposed method. The compliance measure indicates the range of behaviors in which the system operates safely, and through this, the behavior of the system is detected.

The proposed method, SMDFbs, calculates the compliance (*C*) using the following formula after counting the number of times it remained in a normal state (Br) at regular intervals:

(1) $$C_{x}=\frac{Br}{n}$$

After calculating the compliance for the p-th time, the compliance history $C_{1}$, $C_{2}$, $C_{3}$, …, $C_{p}$ is used to make a final determination of whether the state is abnormal using a statistical approach. To utilize the compliance in statistical analysis, the compliance is modeled as a random value following a probability distribution function:

(2) $$G\left(\cdot \right)=B\left(\alpha ,\beta \right)$$

When α is 1, β is set as the compliance history using the maximum likelihood estimation function:

(3) $$\beta =\frac{p}{\sum_{i=1}^{p}log\left(\frac{1}{1-C_{i}}\right)}$$

The average compliance degree is calculated as follows:

(4) $$E\left(C\right)=\frac{1}{1+\beta }$$

If the average compliance is less than the predefined threshold ($C_{T}$), then it is determined to be abnormal:

(5) $$E\left(C\right)<C_{T}$$

## 5. Experiments

### 5.1. Experimental Environment

**Figure 7:**
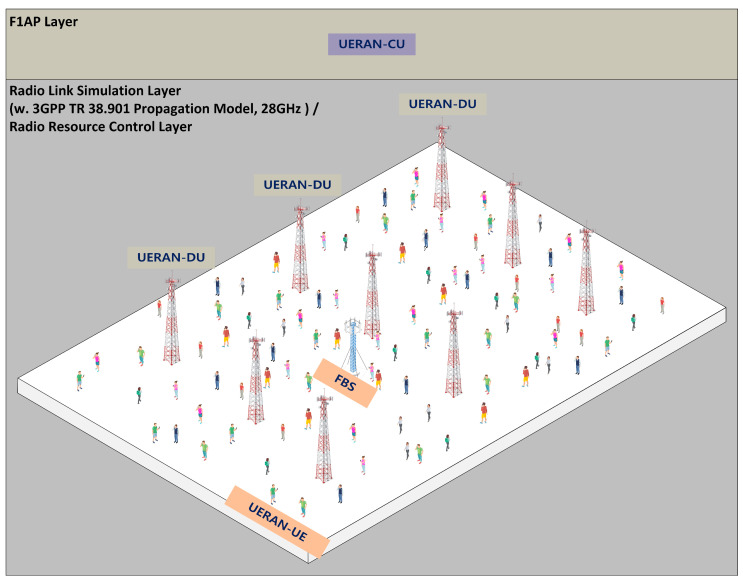
Experimental environment.

Figure 7 represents a schematic of the experimental environment. To establish the experimental environment, a modified version of UERANSIM was employed. UERANSIM is a 5G RAN simulator that supports communication protocol stacks such as gNB and UE’s RRC, Non-Access Stratum (NAS), and others [24]. As UERANSIM lacks the implementation of handover functionality and functional separation, it was necessary to implement essential features for the experiment. Therefore, UERANSIM’s gNB was separated into CU and DU, and the 3GPP’s Propagation Model was applied to implement a 28 Ghz wireless communication environment. Additionally, the Inter gNB-DU handover was implemented, allowing the UE to send a Measurement Report, and the CU was developed to make handover decisions. Experimental data were recorded for a total of one hour, and a scenario where noise occurs for 5 min every 10 min was created. The noise levels were set based on typical noise assessment environments: Normal ([0, 3%]), Low ([0, 10%]), Medium ([0, 20%]), and High ([0, 30%]).

In the simulation, UEs are randomly and uniformly placed within a predefined range, with 100 UEs in total. Every 10 s, they change direction and speed randomly, moving within the set range. Upon reaching the boundaries of the designated range, they reflect like a mirror, changing direction. Each UE is assigned a random seed value between 1 and 100. For instance, the first UE is assigned the random seed value of 1. Furthermore, every 10 min, with a 50% probability, they turn off and turn back on after a minute. If the RRC connection is lost during operation, they search for a new cell.

DUs are fixed at specified coordinates, and each DU is placed in a grid format with a 1000 m distance interval. The DUs relay Uplink RRC messages and Downlink RRC messages to the CU and UEs, respectively. Based on the Uplink RRC messages, the CU processes registration and handover procedures. If an RRC_Setup_Request message is sent again to a previously connected UE, the CU instructs the DU to delete the existing UE Context.

The state machine, written in Python code, operates on preprocessed datasets generated from experiments using the modified UERANSIM, as previously described. The output of this state machine is in the form of Boolean values, representing the condition statements of each behavioral rule. This state machine is executed within the environment where the machine learning experiment is conducted. Subsequently, statistical analysis is performed on the outputs of the state machine for each input dataset.

Fake base station attacks vary in probability and conditions according to their nature. To measure and compare the detection accuracy after performing an attack on a connecting UE, attack data are stored. The fake base station attacker’s tendencies are set as Reckless, Opportunistic, and Hidden, leading to different attack probabilities and conditions.

1. The Reckless attacker acts indiscriminately, generating a random number between [0, 1]. If the number is 0.05 or higher (indicating a 95% attack probability), they initiate the attack. As soon as the scenario begins, they continuously attack throughout its duration. They emit a signal 1.5 times stronger than the surrounding signals to entice nearby UEs to connect.
2. The Opportunistic attacker operates only when noise occurs, reducing the probability of detection. They attack for 5 min every 10 min throughout the scenario’s duration. Like the Reckless attacker, they send out a signal 1.5 times stronger than the surrounding signals to induce nearby UEs to connect.
3. The Hidden attacker generally behaves like the Opportunistic attacker, but with a 20% attack probability. Furthermore, they transmit at the same signal strength as nearby base stations, making detection more challenging.

All attackers, regardless of their disposition, carry out UE_Capability_Information modification, relay, and DoS attacks on UEs attempting to connect, and they record these actions. Detection accuracy is measured based on these records.

### 5.2. Evaluation

The data generated earlier was produced in the environment summarized in Table 2. We compared the proposed specification-based method with seven supervised learning algorithms (SVM, KNN, Decision Tree, Naive Bayes, Random Forest, XGBoost, and Multi-layer perceptron (MLP)). Machine learning was trained using features like message increase amount, request count, etc., aligned with the defined state machine. Each behavioral rule is activated as a single feature for detection. For the test data, the Random Seed was set to 201–300 for UEs.

**Figure 8:**
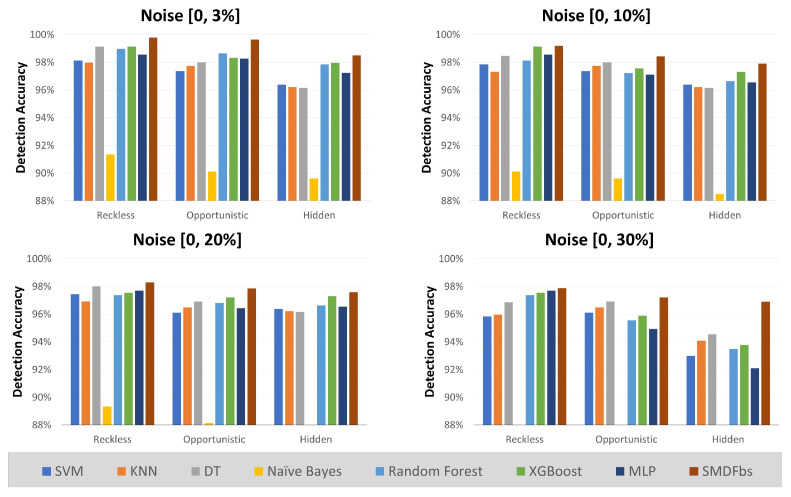
Detection accuracy by noise level.

From the derived behavioral rules, a state machine was constructed. Through iterative testing, an optimal threshold was set, after which the detection accuracy was assessed. Figure 8 illustrates the detection accuracy across different noise levels. The compliance threshold for the specification-based technique in all scenarios of this experiment was set to 0.51. The specification-based technique verified a higher detection accuracy, averaging 98% across all noise levels, outperforming the supervised machine learning algorithms on average. As the noise level increases, the gap in detection performance between specification-based methods and machine learning algorithms widens. Among the supervised learning algorithms, XGBoost exhibited the highest accuracy in detection. The algorithm with the poorest performance was Naïve Bayes. Naïve Bayes is suitable for scenarios where each feature is independent. However, in our case, the features were not entirely independent, which led to these results.

For efficiency evaluation, the proposed detection technique was compared with seven supervised learning algorithms in terms of time and memory usage.

Table 3 presents the computational time and memory consumption of the proposed technique in comparison with other supervised learning algorithms. For machine learning algorithms, the training and test data are partitioned in an 8:2 ratio and are executed as a single dataset. Based on the outcomes, it can be deduced that the proposed approach exhibits superior efficiency in both computational duration and memory utilization relative to alternative methods.

## 6. Conclusions

As we approach the commercialization of 5G and prepare for the future era of 6G, the threat of false base station attacks continues to be an issue, regardless of previous generations of mobile networks. Especially, as 5G’s core features such as enhanced Mobile Broadband (eMBB), Ultra-Reliable Low Latency Communications (URLLC), and massive Machine Type Communications (mMTC) are being utilized for applications like smart factories, Virtual Reality (VR), Augmented Reality (AR), and V2X, these services adhere to security requirements for availability, making them more vulnerable to the impact of false base station attacks, which can lead to Denial of Service. Consequently, continuous research to address these threats has been essential.

Furthermore, due to the operational characteristics of 5G, which utilizes wireless radio technologies covering relatively short ranges, there is a need to deploy an enormous number of radio devices. This high-mobility environment, especially with UEs like vehicles, leads to an increased occurrence of handover events. Consequently, this ultra-dense wireless deployment structure provides malicious attackers with more opportunities to set up small false base stations mimicking legitimate ones, as they can initiate attacks. Successful attacks through false base stations can inflict harm on various C-ITS services, emphasizing the ongoing need for security strategies to counteract these threats.

In this paper, we have adopted a behavior rule specification-based false base station attack detection technique to establish a secure wireless network for the next-generation mobile communication networks. Behavior rule specification-based detection techniques are derived from specification-based detection approaches. These techniques detect attacks based on specified program behavior specifications [22]. They rely on detailed specifications (protocols or behavioral patterns) of normal network operation, treating activities outside of these specifications as intrusions. Typically, such systems employ techniques such as protocol analysis and state machines to detect behaviors which beyond the defined scope. Our proposed behavior rule specification-based detection system generates behavior rules based on UE traffic generated by UE actions during the process of base station processing, taking into account both normal UE behavior rules and those specified in 3GPP standards and security threats. The false base station attack detection system executes this state machine to detect ongoing attacks by false base stations.

To validate the performance of the proposed technique, we improved the 5G system simulation tool, UERANSIM, to align with the characteristics of Functional Split. We conducted experiments in this enhanced environment, generating data for learning and testing. We implemented the SMDFbs false base station detector based on the state machine and compared it to seven supervised learning algorithms using same dataset. The experimental evaluation demonstrated that the proposed technique outperformed machine learning algorithms, achieving an average detection accuracy of 98% across all noise levels. Furthermore, a comparative analysis of time and memory usage indicated that the behavior rule specification-based detection technique incurred minimal overhead, resulting in less strain on computing power.

As a result, machine learning-based techniques exhibited vulnerability to noise and posed a higher overhead burden, making them less suitable for mobile communication convergence services where availability is crucial. Ultimately, this research confirmed that such drawbacks can be complemented by specification-based detection techniques. The behavior rule specification-based detection technique proposed in this study has limitations when it comes to handling complex attacks, as its primary focus is on detecting false base stations.

As a future research direction, we plan to explore detection techniques using generative artificial intelligence to create noise-filtered RSRP values for detection. And we will conduct additional research on the case when multiple state machines are executed in parallel and the environment where multiple false base stations are executed.

## Figures and Tables

**Table 1:** Verification properties via UPPAAL.

Verification Property	Verification Type	Query	Normal	Abnormal
The system does not enter a deadlock	Safety	A[] not deadlock	Satisfied	Unsatisfied
Behavior rules from 1 to 8 were evaluated once.	Reachability	E<>forall (i:id_t)Checker(i).Error	Satisfied	Unsatisfied

**Table 2:** Experimental environment summary.

Environment of Dataset Generation	
Operation System	Ubuntu 22.04-amd64-server LTS
5G RAN Simulator	UERANSIM-LITE
UE	EC2 t2.micro
DU	EC2 t2.micro
CU	EC2 t3.2xlarge
False Base Station	EC2 t2.micro
**State Machine and Machine Learning Test Environment **	
Operation System	Windows 10
CPU	i7-12700KF
RAM	DDR4 64.0 GB
Graphic Card P	GeForce RTX 3070 Ti
Program Language	Python 3.10
ML Library	Scikit-Learn 1.1.2
XGBoost Library	Python XGBOOST 1.7.3

**Table 3:** Overhead (computation, memory usage) by algorithms.

Algorithms	Computation Overhead (ms)	Memory Usage (MB)
Proposed Method	0.820	427
SVM	2321.9216	621
KNN	153.9976	628
Decision Tree	17.4839	625
Naive Bayes	13.0920	619
Random Forest	287.5588	649
XGBoost	520.9858	675
MLP	8142.4864	648

## Data Availability

Publicly available datasets were analyzed in this study. These data can be found here: https://github.com/rjaeka456/UERANSIM-LITE (accessed on 22 October 2023).

## References

[1] SA1 (2023). Service Requirements for the 5G System.

[2] SA3 (2023). Study on 5G Security Enhancements against False Base Stations (FBS).

[3] Karaçay L., Cheng S.M., Kaliski R., Hung C.F. (2021). A Network-Based Positioning Method to Locate False Base Stations. IEEE Access.

[4] SA3 (2023). Security Architecture and Procedures for 5G System.

[5] Chlosta M., Rupprecht D., Pöpper C., Holz T. 5G SUCI-catchers: Still catching them all? In Proceedings of the 14th ACM Conference on Security and Privacy in Wireless and Mobile Networks, Abu Dhabi, United Arab Emirates, 28 June–2 July 2021.

[6] Park H.Y., Kim T.G., Duguma D.G., Kim J., You I., Susilo W. (2023). An Enhanced Group Key-Based Security Protocol to Protect 5G SON Against FBS. Comput. Syst. Sci. Eng..

[7] Nakarmi P.K., Ersoy M.A., Soykan K.E.U. (2021). Norrman, Murat: Multi-rat false base station detector. arXiv.

[8] Nakarmi P.K., Sternby J., Ullah I. Applying Machine Learning on RSRP-based Features for False Base Station Detection. Proceedings of the 17th International Conference on Availability, Reliability and Security.

[9] Jin J., Lian C., Xu M. Rogue Base Station Detection Using A Machine Learning Approach. Proceedings of the 2019 28th Wireless and Optical Communications Conference(WOCC).

[10] Park H., Son D., Kim G., You I. A study on machine learning-based false base station detection method in 5G. Proceedings of the 6th International Symposium on Mobile Internet Security (MobiSec’22).

[11] Ali A., Fischer G. Enabling Fake Base Station Detection through Sample-based Higher Order Noise Statistics. Proceedings of the 2019 42nd International Conference on Telecommunications and Signal Processing (TSP).

[12] RAN 1 (2022). Study on Channel Model for Frequencies from 0.5 to 100 GHz.

[13] RAN 3 (2023). NG-RAN; Architecture Description.

[14] Masini G. (2021). A guide to ng-ran architecture. 5G and Beyond: Fundamentals and Standards.

[15] Bertenyi B., Burbidge R., Masini G., Sirotkin S., Gao Y. (2018). Ng radio access network (ng-ran). J. ICT Stand..

[16] SLS Team (2023). Cell-Site Simulators/IMSI Catchers.

[17] Yomna N. (2019). Gotta Catch’Em All: Understanding How IMSI-Catchers Exploit Cell Networks.

[18] Li Z., Wang W., Wilson C., Chen J., Qian C., Jung T., Zhang L., Liu K., Li X., Liu Y. FBS-Radar: Uncovering Fake Base Stations at Scale in the Wild. Proceedings of the 2017 Network and Distributed System Security Symposium.

[19] Bin Q., Ziwen C., Yong X., Liang H., Sheng S. (2020). Rogue Base Stations Detection for Advanced Metering Infrastructure Based on Signal Strength Clustering. IEEE Access.

[20] Shin J., Shin Y., Park J.G. Network Detection of Fake Base Station using Automatic Neighbour Relation in Self-Organizing Networks. Proceedings of the 2022 13th International Conference on Information and Communication Technology Convergence (ICTC).

[21] Sharma V., You I., Yim K., Chen I.R., Cho J.H. (2019). BRIoT: Behavior Rule Specification-Based Misbehavior Detection for IoT-Embedded Cyber-Physical Systems. IEEE Access.

[22] Astillo P.V., Choudhary G., Duguma D.G., Kim J., You I. (2021). TrMAps: Trust Management in Specification-Based Misbehavior Detection System for IMD-Enabled Artificial Pancreas System. IEEE J. Biomed. Health Inform..

[23] UPPAAL: Integrated Tool Environment for Modeling, Validation and Verification. http://uppaal.org.

[24] UERANSIM. https://github.com/aligungr/UERANSIM.

